# A Real-world Data Analysis of Intermittent Catheterization, Showing the Impact of Prelubricated Versus Hydrophilic Catheter Use on the Occurrence of Symptoms Suggestive of Urinary Tract Infections

**DOI:** 10.1016/j.euros.2022.02.008

**Published:** 2022-03-04

**Authors:** Emmanuel Chartier-Kastler, Christopher Chapple, Brigitte Schurch, Mehdi Saad

**Affiliations:** a Department of Urology, Médecine Sorbonne Université, Pitié-Salpêtrière Academic Hospital, Assistance Publique-Hôpitaux de Paris, Paris, France; b Department of Urology, Royal Hallamshire Hospital, Sheffield Teaching Hospitals NHS Trust, Sheffield, UK; c Neuro-urology Unit, Department of Clinical Neurosciences, Vaudois University Hospital of Lausanne and University of Lausanne, Lausanne, Switzerland; d B. Braun Medical SAS, Saint-Cloud, France

**Keywords:** Urinary tract infection, Intermittent urinary catheter, Hydrophilic, Prelubricated, Propensity score matching

## Abstract

**Background:**

Systematic reviews have highlighted the lack of evidence on choosing the type of intermittent urinary catheter (IUC) with regard to the occurrence of urinary tract infections (UTIs).

**Objective:**

To describe the incidence and frequency of symptoms suggestive of UTIs (ssUTIs) for prelubricated versus hydrophilic IUCs.

**Design, setting, and participants:**

An observational study of a patient database compiled by UK general practitioners was conducted.

**Outcome measurements and statistical analysis:**

The primary outcome measures were the proportion of patients with at least one ssUTI (prescription of a nonspecific antibiotic with a UTI-related diagnosis, or prescription of a UTI-specific antibiotic) and the mean number of ssUTIs per affected patient in the 12 mo following the index IUC prescription. Comparable prelubricated (“PRELUBE”) and hydrophilic (“HYDRO”) catheter groups were obtained with 1:1 propensity score matching (PSM).

**Results and limitations:**

A total of 5296 patients were included (prelubricated: *n* = 458; hydrophilic: *n* = 4838). After PSM, the two groups had similar proportions of patients with ssUTIs at baseline. The proportion of patients with ssUTIs during exposure was similar in the PRELUBE (36.9%) and HYDRO groups (41.5%; *p* = 0.155). However, among patients having used the same type of catheter throughout the exposure period, the proportion with ssUTIs was significantly lower in the PRELUBE group (44.6%, vs 55.0% for HYDRO; *p* = 0.015), as was the number of ssUTIs per patient (1.3 vs 1.8; *p* = 0.036).

**Conclusions:**

When choosing a coated IUC, physicians and patients should not rule out PRELUBE IUCs for safety reasons alone.

**Patient summary:**

Using real-world data compiled by UK general practitioners, we described the incidence and frequency of symptoms suggestive of urinary tract infection in people who were using various types of intermittent urinary catheters. When the same type of prelubricated catheter was used throughout the study period, the incidence of these symptoms was lower than for hydrophilic catheters.

## Take Home Message

In a real-world data analysis of routine clinical practice, coated intermittent urinary catheters (IUCs) showed a good safety profile. Overall, the results for prelubricated (PRELUBE) and hydrophilic (HYDRO) IUCs were similar. However, when considering patients who used the same type of catheter throughout the study, PRELUBE IUCs were associated with a significantly lower mean number of symptoms suggestive of urinary tract infections. When choosing a coated IUC, physicians and patients should not rule out PRELUBE IUCs for safety reasons alone.

## 1 Introduction

Urinary retention is an important health issue [1], [2], [3]. According to European and US guidelines, the first-line treatment option for chronic urinary retention is intermittent self- or heterocatheterization with single-use intermittent urinary catheters (IUCs) [4], [5]. Known risk factors for urinary tract infections (UTIs) in IUC users include low catheterization frequency, bladder overdistension, female sex, inadequate fluid intake, poor technique, and absence of catheter lubrication [6], [7]. In the literature, the number of suspected or proven UTIs among the users of IUCs varies greatly from one clinical study to another (from 0.13 to over 0.68 per month) [8], [9].

Coated catheters (prelubricated or hydrophilic) appear to be the best option for safe, long-term intermittent catheterization [10], [11], [12], [13]. However, literature data on the relative safety of the various types of coated IUCs are scarce. Systematic reviews and best practice reports have highlighted the lack of robust evidence for the superiority of one type of catheter or technique over another [13]. Furthermore, UTIs have been defined in different ways: clinical signs or symptoms, antibiotic prescription, positive urine culture with pyuria, etc. [8], [14]. Rognoni and Tarricone’s [8] meta-analysis of six randomized clinical trials (RCTs) found that the relative risk of a UTI (defined as clinical symptoms and/or culture results) was 0.84 (0.75–0.94) for hydrophilic catheters, relative to nonhydrophilic catheters.

The lack of “gold standard” RCT data means that it is difficult to provide guidance to clinicians or users on techniques and devices. We therefore analyzed real-world data (RWD) on routine IUC use by patients. Real-world analyses take account of patient characteristics, lifestyle factors, treatment compliance, regimens, comorbidities, and quality of life [15], [16], [17].

By analyzing a longitudinal patient database (LPD) compiled by 1950 general practitioners (GPs) in the UK (414 of whom prescribed IUCs), we compare prelubricated IUCs (primarily the Actreen product range from B.Braun Medical SAS, Saint-Cloud, France) and hydrophilic IUCs (primarily the SpeediCath product range from Coloplast A/S, Humlebæk, Denmark) with regard to the incidence and frequency of symptoms suggestive of UTIs (ssUTIs).

## 2 Patients and methods

### 2.1 Study design and database

We performed a retrospective, longitudinal, RWD analysis with propensity score matching (PSM). The anonymized data were extracted from a UK LPD (IQVIA, London, UK) [18]. The LPD’s data on demographic, clinical, and laboratory variables come from electronic health records entered by a nationally representative panel of 1950 GPs and 2 950 381 active patients (in 2017), using proprietary practice management software. The data are cleaned and blinded by IQVIA’s data center. Each visit or prescription is associated with an anonymized patient identifier, and individual patient histories can be tracked.

### 2.2 Procedures

**Fig. 1:**
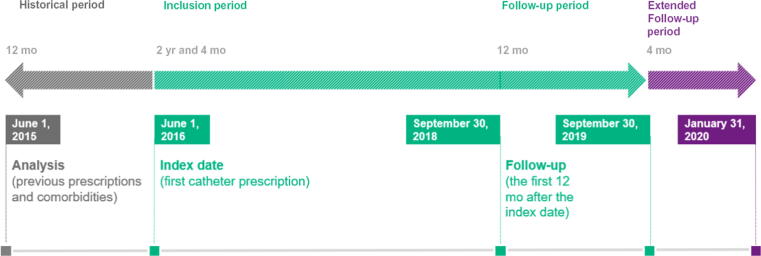
Study timeline. The study comprised four periods: a 12-mo preindex period, a 28-mo inclusion period (comprising the index date), a main (12-mo) follow-up period, and an extended (4-mo) follow-up period.

The analysis comprised four periods: a 12-mo preindex period, a 28-mo inclusion period (comprising the index date), a main (12-mo) follow-up period, and an extended (4-mo) follow-up period (Fig. 1).

The preindex period was defined as the 12 mo preceding the index date; it provided a reliable description of the patient’s baseline characteristics and any previous initiation of catheter use. The inclusion period ran from June 1, 2016, to September 30, 2018. The index date was defined as the date of the first catheter prescription during the inclusion period. The main follow-up period was defined as the 12 mo following the index date; catheter prescriptions (ie, exposure) and study outcomes were calculated for all or part of the main follow-up period. The extended follow-up period was defined as the 4 mo following the main follow-up period; this period enabled us to confirm that a given patient was still using IUCs and was not lost to follow-up (eg, deceased).

### 2.3 Inclusion and exclusion criteria

The inclusion criteria were age 18 yr or over at the index date, at least one prescription of a prelubricated or hydrophilic catheter during the inclusion period, a preindex period of at least 1 yr, and data available for the main and extended follow-up periods. Incident and prevalent patients were, respectively, defined as those without and with a catheter prescription during the preindex periods. The exclusion criteria were as follows: (1) the prescription of two or more different types of catheters at the index date, and (2) the absence of a visit to the GP (ie, no follow-up data) during the follow-up period and the extended follow-up period.

### 2.4 Assessment of ssUTIs

The patients’ event dates and diagnoses were mapped to the UK national thesaurus and then the International Classification of Diseases, 10th revision (ICD-10) [19], and prescription information was mapped to the Anatomical Therapeutic Chemical (ATC) classification [20].

Given the lack of urine culture data in the study database, ssUTIs were defined as either (1) the prescription of an antibiotic whose sole indication is a UTI (based on the European guidelines [4]) or (2) the prescription of a nonspecific antibiotic (ATC code) with a diagnosis (ICD-10 code) related to a UTI (Supplementary Table 1). If two ssUTIs were detected in the same patient within a 4-wk period, those were considered to be a single event.

### 2.5 Outcomes

The primary outcome measure was the occurrence of at least one ssUTI during the main follow-up period. Once patients with ssUTIs had been identified, the mean number of ssUTIs per affected patient was also calculated. The secondary outcome measures included the duration of use, daily frequency of use, and switch rate. All outcomes were analyzed for the study groups as a whole (prelubricated IUCs vs hydrophilic IUCs) and for various subpopulations (incident vs prevalent IUC use, indication for IUC use, by age, by sex, etc.).

### 2.6 Propensity score matching

Given the study’s nonrandomized design, we used 1:1 PSM with multivariable logistic regression adjustment, greedy matching (calliper: 0.25), and stratification (Supplementary material) [21], [22], [23], [24] to compose two patient groups with essentially identical characteristics at the index date. In summary, a patient from the smaller group was selected and then paired with a patient from the larger group with the closest propensity score. This process was repeated until all the patients in the smaller group had been matched.

### 2.7 Statistical analysis

Continuous variables were described as the number of valid cases, number of missing values, mean, standard deviation (SD), median, interquartile range, and range. The normality hypothesis was probed with the Shapiro-Wilk test. Categorical variables were described as the frequency (percentage). Missing values were excluded from the calculation of percentages. All analyses were performed using SAS software (SAS Enterprise Guide 6 or SAS version 9.4; SAS Institute, Inc., Cary, NC, USA). The threshold for statistical significance was set to *p* < 0.05 in general and *p* < 0.2 for the selection of variables for PSM. We applied a chi-square test or Fisher’s exact test for categorical variables, or the Student *t* test, Mann-Whitney test, or Kruskal-Wallis test for continuous variables. Analyses were stratified by incident/prevalent status, age group, sex, and patient status at the end of the main follow-up period: complete discontinuation (no further catheter prescriptions), a switch in the type of catheter (eg, from hydrophilic to prelubricated), or continuous exposure (use of the same catheter type throughout the main follow-up period).

## 3 Results

### 3.1 Study groups before PSM

**Fig. 2:**
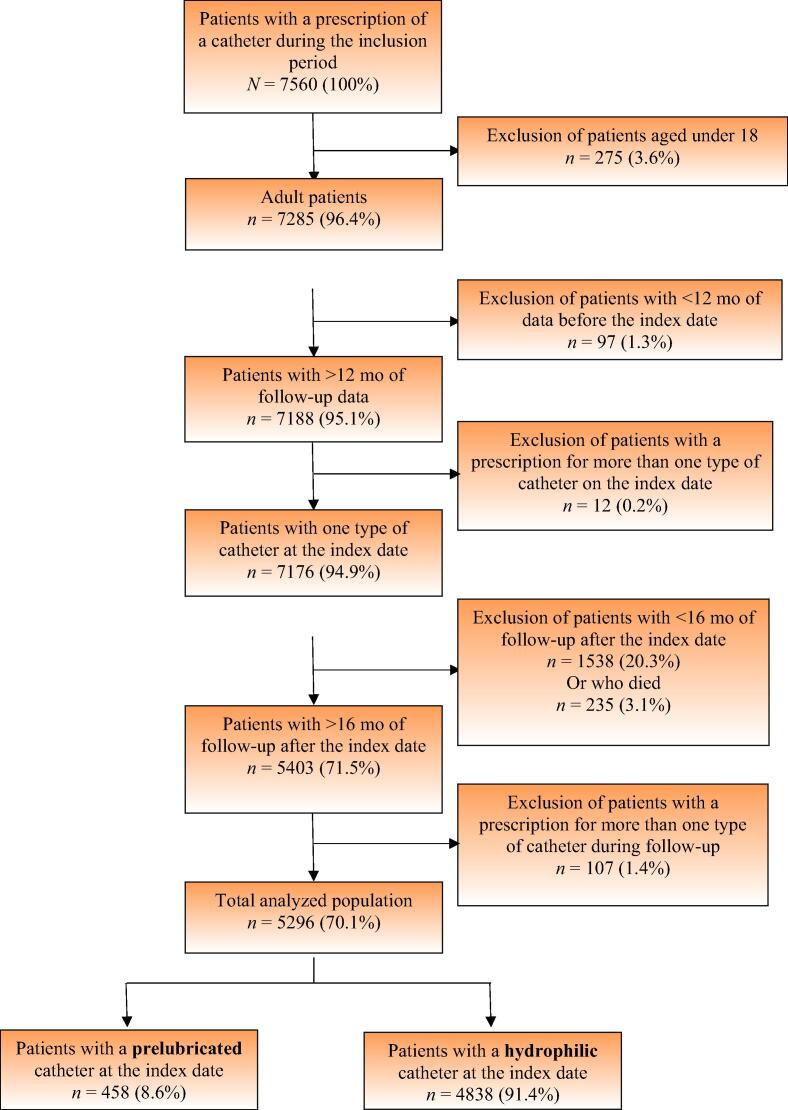
Patient disposition.

The retrospective data covered the period from June 1, 2015, to January 31, 2020. After applying the inclusion and exclusion criteria, data for 5296 patients were included: 458 (8.6%) with a first prescription of a prelubricated catheter at the index date (the “PRELUBE” group) and 4838 (91.4%) with a first prescription of a hydrophilic catheter (the “HYDRO” group; Table 1 and Fig. 2). Just under half of the patients had not used an IUC before the index date and were therefore incident patients. Of the prevalent patients, 70% had been using IUCs for at least a year.

**Table 1:** Characteristics of the two study groups before and after PSM

		Before PSM			After PSM	
		Prelubricated catheter (*N* = 458)	Hydrophilic catheter (*N* = 4838)	*p* value	Hydrophilic catheter (*N* = 458)	*p* value
Age (yr)	*N* (%)	458 (100)	4838 (100)		458 (100)	
	Mean (SD)	58.0 (17.55)	62.3 (16.25)	<0.001	57.9 (16.92)	0.931
	Median (IQR)	60.0 (46.0–72.0)	65.0 (51.0–75.0)		58.0 (46.0–72.0)	
	Range	(18.0, 94.0)	(18.0, 100.0)		(18.0, 93.0)	
Age (yr), classes, *N* (%)	18–50	148 (32.3)	1070 (22.1)	<0.001	148 (32.3)	0.948
	50–70	177 (38.6)	1868 (38.6)		181 (39.5)	
	≥70	133 (29.0)	1900 (39.3)		129 (28.2)	
Sex, *N* (%)	Male	186 (40.6)	3154 (65.2)	<0.001	189 (41.3)	0.840
	Female	272 (59.4)	1684 (34.8)		269 (58.7)	
Region of residence, *N* (%)	England	70 (15.3)	1309 (27.1)	<0.001	69 (15.1)	0.987
	London, England	86 (18.8)	395 (8.2)		89 (19.4)	
	Northern Ireland	86 (18.8)	506 (10.5)		88 (19.2)	
	Scotland	182 (39.7)	1585 (32.8)		175 (38.2)	
	Wales	34 (7.4)	1043 (21.6)		37 (8.1)	
BMI—quantitative variable (kg/m^2^) a	*N* (%)	193 (42.1)	2079 (43.0)		191 (41.7)	
	Mean (SD)	28.3 (6.50)	28.1 (5.83)	0.644	27.8 (6.08)	0.440
	Median (IQR)	27.1 (23.8–31.9)	27.3 (24.2–31.3)		26.6 (23.5–31.9)	
	Range	(16.7, 56.8)	(11.2, 62.5)		(16.4, 47.5)	
	Missing, *N* (%)	265 (57.9)	2759 (57.0)		267 (58.3)	
BMI classes (kg/m^2^) a*N* (%)	Underweight <18	3 (0.7)	34 (0.7)	0.927	5 (1.1)	0.829
	Normal weight (18–25)	62 (13.5)	612 (12.6)		65 (14.2)	
	Overweight (25–30)	65 (14.2)	754 (15.6)		55 (12.0)	
	Obesity ≥30	63 (13.8)	679 (14.0)		66 (14.4)	
	Missing	265 (57.9)	2759 (57.0)		267 (58.3)	
At least one pathology leading to intermittent catheterization		288 (62.9)	3009 (62.2)	0.772	283 (61.8)	0.733
Neurourological dysfunctions, *N* (%)	All types	69 (15.1)	554 (11.5)	0.026	69 (15.1)	>0.999
	Equina syndrome	7 (1.5)	17 (0.4)	0.004	1 (0.2)	0.024
	Multiple sclerosis	24 (5.2)	115 (2.4)	0.001	20 (4.4)	0.536
	Neuropathy	11 (2.4)	77 (1.6)	0.221	14 (3.1)	0.542
	Spinal cord injury	5 (1.1)	71 (1.5)	0.501	6 (1.3)	0.761
	Others a	25 (5.5)	287 (5.9)	0.677	30 (6.6)	0.487
Consequences of other surgical operations, *N* (%)		36 (7.9)	346 (7.2)	0.580	31 (6.8)	0.526
Bladder issues b*N* (%)		14 (3.1)	262 (5.4)	0.020	13 (2.8)	0.845
Other urinary tract disorders c*N* (%)		224 (48.9)	2347 (48.5)	0.871	224 (48.9)	>0.999
Genital system disorders d*N* (%)		74 (16.2)	916 (18.9)	0.139	83 (18.1)	0.430
Catheter status at index date (whatever the type of catheter), *N* (%)	Incident	226 (49.3)	2362 (48.8)	0.831	224 (48.9)	0.895
	Prevalent	232 (50.7)	2476 (51.2)		234 (51.1)	
Diabetes (ICD-10 E10-E14, O24), *N* (%)		15 (3.3)	150 (3.1)	0.838	19 (4.1)	0.484
At least one ssUTI in the preindex period, *N* (%)		215 (46.9)	2130 (44.0)	0.230	220 (48.0)	0.741
End of follow-up status, *N* (%)	Continuous	269 (58.7)	2915 (60.3)		280 (61.1)	
	Stop	170 (37.1)	1894 (39.1)		176 (38.4)	
	Switch	19 (4.1)	29 (0.6)	<0.001	2 (0.4)	<0.001

BMI = body mass index; ICD-10 = International Classification of Diseases, 10th revision; IQR = interquartile range; PSM = propensity score matching; SD = standard deviation; ssUTI = symptoms suggestive of urinary tract infection.

a Other neurourological dysfunctions included cerebellar hemorrhage, stroke, transient ischemic attack, motor symptoms not otherwise specified (NOS), Guillain-Barre syndrome, Parkinson’s disease, paralysis NOS, syringomyelia, myelopathy NOS, malignant neoplasm of the lumbar vertebrae, hemiparesis, and congenital cerebral palsy.

b Bladder issues included atonic bladder, hypotonic bladder, bladder neck obstruction, congenital bladder neck obstruction, neuromuscular dysfunction of the bladder, in situ carcinoma of the bladder, bladder diverticulitis, stenosis of the bladder neck, malignant neoplasm of the bladder, urethra or bladder neck atresia or stenosis NOS, squamous metaplasia of the bladder, and overactive bladder.

c Other urinary tract disorders included retention of urine, chronic retention of urine, incontinence of urine, urgency of micturition, vesical pain, urothelial carcinoma, malignant neoplasm of urinary organ, unspecified, epididymitis, malignant neoplasm of ureter, nocturia, nocturnal enuresis, orchitis and epididymitis, rectal prolapse, malignant neoplasm of rectum, and malignant neoplasm of the body of the penis.

d Other genital system disorders included prostatectomy, prostatic hyperplasia, acute prostatitis, prostatocystitis, adenoma of the prostate, neoplasm of the prostate, in situ carcinoma of the prostate, prostate cancer, enlarged prostate, genital prolapse, hysterectomy, orchidectomy, malignant neoplasm of endometrium, malignant neoplasm of the testis, and menopausal and postmenopausal disorders.

### 3.2 Study groups after PSM

The following variables were included in a multivariable logistic regression model of the propensity score: age (*p* < 0.001), sex (*p* < 0.001), region of residence (*p* < 0.001), frequency of switching (*p* < 0.001), the main indication for IUCs (*p* = 0.014), and the number of ssUTIs experienced during the preindex period (*p* = 0.08; Table 2). Although the intergroup difference in incident versus prevalent status was not significant (*p* = 0.8), this important safety variable was also included in the PSM.

**Table 2:** Variables selected for inclusion in a logistic regression model of the propensity score for matching prelubricated versus hydrophilic IUC users

Variable	Classes	*p* value a
*Patient profile at the index date*		
Age (yr), classes	18–49, 50–69, ≥70	<0.0001
Sex	Male, female	<0.0001
Region	London, England, Northern Ireland, Scotland, Wales	<0.0001
*Indication in the 2 yr before the index date or in the year after the index date*		
Main disease or disorder leading to intermittent catheterization	Neurourological dysfunctions, bladder issues, other urinary tract disorders, consequences of other surgical operations, genital system disorders, none of the above	0.0136
*Past catheter use*		
Catheter use status (whatever the type) at the index date	Incident/prevalent	0.8305
*Comorbidities and risk factor for UTI in the year before index date*		
Number of UTIs during the year before index date	0, 1–2, ≥3	0.0816

IUC = intermittent urinary catheter; UTI = urinary tract infection.

a In a univariate logistic regression.

**Fig. 3:**
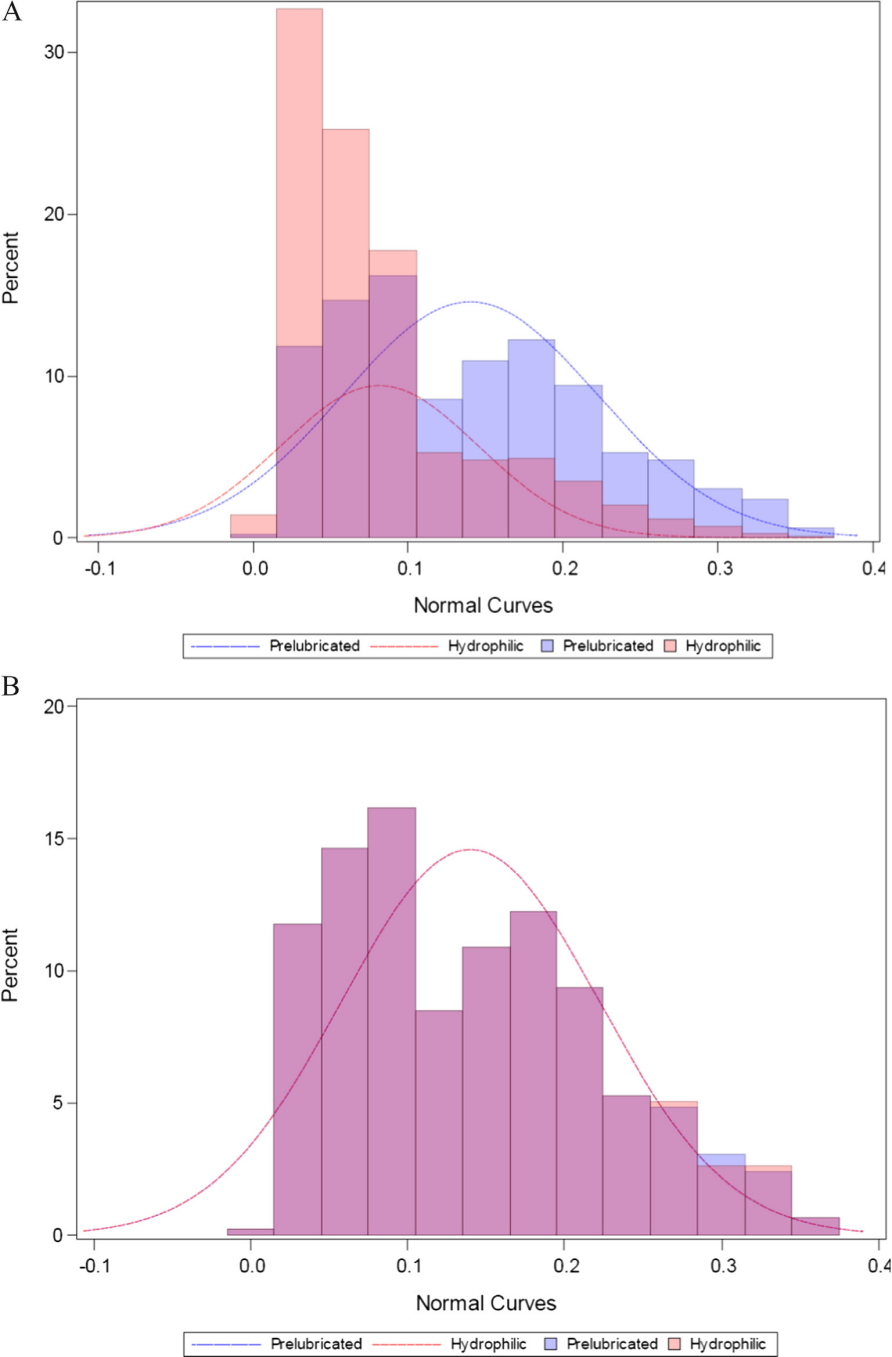
The propensity score distributions (A) before and (B) after PSM in the PRELUBE and HYDRO groups. HYDRO = hydrophilic catheter; PRELUBE = prelubricated catheter; PSM = propensity score matching.

Each of the 458 prelubricated catheter users was matched with a hydrophilic catheter user (Table 1), giving 916 users in all (females: *n* = 541, 59.1%; mean ± SD age: 58 ± 17). Figure 3 shows the propensity score distribution in the PRELUBE and HYDRO groups before and after PSM. After PSM, there were no statistically significant differences between the PRELUBE and HYDRO groups with regard to the score’s variables (Table 1 and Fig. 3). Henceforth, all results quoted for the HYDRO group refer to the group after PSM.

### 3.3 Primary outcome: incidence of ssUTIs

The proportions of patients having experienced at least one ssUTI during the 12-mo follow-up period were similar in the PRELUBE versus HYDRO group (36.9% vs 41.5%; *p* = 0.155; Table 3). However, when considering the “continuous use” subpopulation, the proportion of patients with at least one ssUTI was significantly lower in the PRELUBE group than in the HYDRO group (44.6% and 55.0%, respectively; *p* = 0.015).

**Table 3:** Primary outcome: the proportion of patients with at least one ssUTI during the main (12-mo) follow-up period, after PSM

Population	Prelubricated catheter group	Hydrophilic catheter group	*p* value
Overall	36.9% (169/458)	41.5% (190/458)	0.155
Continuous use	44.6% (120/269)	55.0% (154/280)	**0.015**
Stop	24.1% (41/170)	20.5% (36/176)	0.413
Switch	42.1% (8/19)	0% (0/2)	Not applicable a
Males	34.9% (65/186)	37.0% (70/189)	0.673
Females	38.2% (104/272)	44.6% (120/269)	0.132
Age group 18–49 yr	33.1% (49/148)	39.9% (59/148)	0.227
Age group 50–69 yr	41.8% (74/177)	40.9% (74/181)	0.859
Age group ≥70 yr	34.6% (46/133)	44.2% (57/129)	0.111

PSM = propensity score matching; ssUTI = symptoms suggestive of urinary tract infection.

a The sample size was too small for a statistically robust comparison.

In both groups, the proportion of women with at least one ssUTI was slightly but nonsignificantly higher than the corresponding proportion of men. Age class was not significantly associated with having at least one ssUTI.

Among incident patients, the mean time to the first ssUTI during the main follow-up period was longer (albeit not significantly) in the PRELUBE group than in the HYDRO group (100.1 and 84.3 d, respectively; *p* = 0.299).

### 3.4 Primary outcome: mean number of ssUTIs during exposure

When considering the number of ssUTIs per affected patient, the overall mean number during the exposure period was significantly lower in the PRELUBE group than in the HYDRO group (0.9 vs 1.3, considering all the 458 patients in each matched group; *p* = 0.006). This difference in the mean number was also significant when considering (1) only patients with at least one ssUTI (*n* = 169 and *n* = 190 in the PRELUBE and HYDRO groups, respectively; 2.5 vs 3.0 ssUTIs; *p* = 0.013), (2) the “continuous” population only (*n* = 269 and *n* = 280, respectively; 1.3 vs 1.8 ssUTIs; *p* = 0.004), (3) women (*n* = 272 and *n* = 269, respectively; 1.0 vs 1.4 ssUTIs; *p* = 0.010), (4) prevalent patients (*n* = 232 and *n* = 234, respectively; 1.2 vs 1.6 ssUTIs; *p* = 0.031), and the 18–49 yr age class (*n* = 148 and *n* = 148, respectively; 0.7 vs 1.2 ssUTIs; *p* = 0.021).

### 3.5 Secondary outcomes

With regard to the duration of use, the time interval from the index date to the stop date was similar in the PRELUBE and HYDRO groups (95.3 vs 109.3 d, *p* = 0.250). The mean number of catheters used per day during the exposure period was essentially the same in the PRELUBE and HYDRO groups (2.8 for men and 2.9 for women), but was slightly and significantly higher in the PRELUBE group when considering incident patients (2.7, vs 2.3 in the HYDRO group; *p* = 0.046). Switches from prelubricated IUCs to hydrophilic IUCs (19 out of 458 [4.1%]) and from hydrophilic IUCs to prelubricated IUCs (two out of 458 [0.4%]) at study end were both very infrequent, and the small sample sizes precluded intergroup statistical comparisons (Table 3).

## 4 Discussion

In order to study users of prelubricated versus hydrophilic IUCs, we used PSM to form comparable PRELUBE and HYDRO groups (predominantly middle-aged/older adults, with female predominance and a third classified as overweight or obese). It is noteworthy that during the exposure period, the mean daily consumption was around three catheters (2.8–3.1) in both groups; this is lower than recommended in the guidelines, even though the LPD’s GP panel is reportedly representative of UK practices. There are several possible explanations for this difference with the guidelines. First, the number of catheters prescribed is not restricted. Second, prescribing practice varies from one region to another, but most local payers follow national guidelines [25]. Third, some patients are still able to partly void their bladder through miction and so may not change their IUC five times a day [26]. Hence, the low consumption is likely to reflect routine clinical practice, rather than limited prescription by GPs. Importantly, these factors would probably affect PRELUBE and HYDRO users to the same extent.

After PSM, the PRELUBE versus HYDRO difference in the proportion of patients with at least one ssUTI was not significant (36.9% vs 41.5%; *p* = 0.155). This finding constitutes robust evidence of similar levels of safety in prelubricated and hydrophilic IUCs. However, when comparing patients in the matched PRELUBE and HYDRO groups who used the same type of catheter throughout the exposure period, the mean number of ssUTIs per 12 mo was significantly lower in the PRELUBE group (0.9) than in the HYDRO group (1.3). The use of prelubricated catheters was still linked to a lower occurrence of ssUTIs when considering vulnerable patient populations (women and prevalent patients).

The present study of an LPD had several strengths. First, it constituted the first analysis of RWD on the clinical safety of prelubricated and hydrophilic IUCs. RWD and RCT data are considered mutually complementary, bearing in mind the different facets of both types of evidence, and realizing that RWD are based on the sample taken but nevertheless provide strong clinical applicability of RCT data [17]. Although RCTs will always be the gold standard for evaluating efficacy and safety, it is rarely possible to recruit a study population of several thousand community-dwelling patients over a short period of time. In contrast, RWD on effectiveness and safety can be gathered quickly once the extraction protocol has been configured. Patient populations, treatment patterns, follow-up monitoring, and comparator interventions are necessarily more heterogeneous in real-world studies than in RCTs [17]. The LPD analyzed in the present study reflects real-world IUC use and can provide alternative markers of safety. It is noteworthy that the data in the LPD were collected in an unbiased, noninterventional way and so reflect routine clinical practice in the GPs’ surgeries. The data were entered during routine patient care and submitted on a regular basis to the coordinating center for cleaning and deidentification. This method of data collection enabled an a posteriori analysis of the patient’s entire prescription and care history. Second, the study looked at pragmatic primary care management in the community by a representative panel of UK GPs and patients. Last, the proportions of users of prelubricated versus hydrophilic IUCs observed in the database reflect the UK sales data in 2020 [25].

The study also had a number of limitations, many of which were linked to its retrospective design and the use of prescription data (rather than clinical data). First, we did not have access to data on urine cultures or other reliable methods of attesting to a UTI, and could not confirm the presence of a bacteriologically confirmed UTI on the basis of the patient’s LPD data. For example, our selected ICD codes would probably not have reliably identified cases of asymptomatic bacteriuria and so might have led to under-reporting of UTIs. However, antibiotics are often inappropriately prescribed when asymptomatic bacteriuria is suggested, leading to an overestimation of the frequency of UTIs [27]. Hence, we chose to refer to “symptoms suggestive of a UTI” on the basis of symptom-based diagnostic codes and antibiotic prescription data. Despite its disadvantages, this approach corresponds to real-life clinical practice because the diagnosis in primary care is not always confirmed by a gold standard urine culture. Furthermore, we expect that any bias in the diagnosis and treatment of asymptomatic bacteriuria would affect users of prelubricated and hydrophilic IUCs to the same extent. It has also been reported that ICD-10 diagnosis codes constitute a valid method for studying UTIs in primary care settings [28]. Second, anonymization of the patient data prevented us from querying the GPs' records; hence, there was a risk of under-reporting. Third, a selection bias was possible because data on patients managed by specialist physicians and/or in hospital were not collected. Accordingly, we included an extended follow-up period to ensure that the patient was still seeing his/her GP, and that his/her final status (“discontinuation”, “stop”, “switch”, or “continuous”) could be determined. Fourth, the use of PSM always represents a tradeoff between bias reduction on one hand and precision on the other [23], [24]. Fifth, it is possible that the administration of certain medications (eg, analgesics) masked some episodes. However, it is unlikely that this type of medication use would differ significantly between matched PRELUBE and HYDRO users. Although a poor self-catheterization technique per se is well known to be associated with a greater frequency of UTIs, the two types of UTIs have very similar gross characteristics and self-catheterization techniques, and so a difference between the PRELUBE and HYDRO groups is unlikely. Last, the study was limited to an LPD from the UK; it would be interesting to perform similar studies in countries or regions with different healthcare, social care, and reimbursement systems.

Despite these limitations, we consider that an RWD analysis was perfectly relevant for measuring product effectiveness and safety in routine clinical practice. A patient and his/her GP are likely to consider many criteria when selecting a suitable coated IUC: design, compactness, ease of use, comfort, brand awareness, cost, and reimbursement; a holistic approach should be adopted. Cost is unlikely to have been a confounding factor in the present study. In the UK, IUCs are fully reimbursed for a large proportion of patients. Even when a patient is not exempted, the reimbursement level and thus the cost are similar for the PRELUBE and HYDRO IUCs. Likewise, GPs in the UK do not have financial reasons for preferring PRELUBE IUCs to HYDRO IUCs or vice versa.

Lastly, our results raise a number of important questions. What would be the results of similar studies in groups of patients with bacteriologically confirmed UTIs? Moreover, what are the characteristics of particular subtypes of prelubricated and hydrophilic catheters?

## 5 Conclusions

In an RWD analysis of routine clinical practice, coated IUCs showed a good safety profile. Overall, the results for PRELUBE and HYDRO IUCs were similar; hence, when choosing a coated IUC, physicians and patients should not rule out PRELUBE IUCs for safety reasons. Indeed, when considering patients who used the same type of catheter throughout the study, PRELUBE IUCs were even associated with a significantly lower mean number of ssUTIs.

  ***Author contributions*:** Emmanuel Chartier Kastler had full access to all the data in the study and takes responsibility for the integrity of the data and the accuracy of the data analysis.

*Study concept and design*: Chartier Kastler, Saad.

*Acquisition of data*: Chartier Kastler, Saad.

*Analysis and interpretation of data*: Chartier Kastler, Chapple, Schurch, Saad.

*Drafting of the manuscript*: Chartier Kastler, Chapple, Schurch, Saad.

*Critical revision of the manuscript for important intellectual content*: Chartier Kastler, Chapple, Schurch, Saad.

*Statistical analysis*: Chartier Kastler, Saad.

*Obtaining funding*: Chartier Kastler.

*Administrative, technical, or material support*: Chartier Kastler, Saad.

*Supervision*: Chartier Kastler, Saad.

*Other*: None.

  ***Financial disclosures*:** Emmanuel Chartier Kastler certifies that all conflicts of interest, including specific financial interests and relationships and affiliations relevant to the subject matter or materials discussed in the manuscript (eg, employment/affiliation, grants or funding, consultancies, honoraria, stock ownership or options, expert testimony, royalties, or patents filed, received, or pending), are the following: Emmanuel Chartier Kastler is a consultant, investigator, or speaker for Allergan, Astellas, B. Braun Medical SAS, Boston Scientific, Coloplast, Convatec, Medtronic, and Uromems. Christopher Chapple is a consultant, investigator, or speaker for Allergan, Astellas Pharma, Bayer Schering Parma AG, B. Braun Medical SAS, Contura, Ferring, Poesis Medical, Symimetic, Takeda, and Urovant Sciences. Brigitte Schurch has no conflicts of interest. Mehdi Saad is an employee of B.Braun Medical SAS.

  ***Funding/Support and role of the sponsor*:** The study was funded by B.Braun Medical (Saint-Cloud, France). The funding party did not influence the interpretation or reporting of the present research.
