# Supplementary material for: A Real-world Data Analysis of Intermittent Catheterization, Showing the Impact of Prelubricated Versus Hydrophilic Catheter Use on the Occurrence of Symptoms Suggestive of Urinary Tract Infections
Source: Eur Urol Open Sci. 2022 Mar 4;38:79–87. doi: 10.1016/j.euros.2022.02.008 (PMC9051966; doi:10.1016/j.euros.2022.02.008)
Supplement: Supplementary Data 1 [file mmc1.docx]

Supplementary material

*The propensity score*

Regression adjustment, matching, and stratification using propensity scores are widely used techniques to compare groups - usually a treatment group vs. a non-treatment group. The matching technique based on propensity score is conducted to build groups of patients with the same characteristics. In the present study, we used propensity score matching (PSM) to create groups with the same characteristics for prelubricated catheters vs. hydrophilic catheters. Propensity scores can be described as the conditional probability that a patient receives one of the catheter type given the observed covariates at index date. The propensity score was calculated using all available and relevant covariates and checked for balance.

Histograms of propensity scores distribution before and after PSM were used to visualize the quality of matching. A good degree of overlap shows that prelubricated catheters and hydrophilic catheters were not prescribed to radically different respective patient populations and that PSM created two equivalent populations. On the variable level, the absolute standardized difference was used after matching, and variables with a value higher than 0.10 were judged to be “not balanced” in the PSM and so were included as covariates in the subsequent analysis [21, 22].

Logistic regression

In order to create the propensity score, the following variables were included in the logistic model:

- the patient profile at index date.
- the patient’s indications in the year before the index date.
- past catheter usage in the year before the index date.
- and risk factor for UTI in the year before the index date.

In order to build the propensity score, a logistic regression was implemented. Several steps must be followed to obtain the correct model:

- Study of the form and distribution of the criterion of interest Y to be explained (being treated with a prelubricated catheter vs. a hydrophilic catheter).
- Finalisation and validation of the factors likely to explain the criterion Y that one seeks to model. Some factors may be reworked by the creation of indicator variables i.e., binary or *ad hoc* classifications.
- Detection in a bivariate analysis of the significant relationship between criterion Y (possibly transformed/discretised) and each of the factors identified as potentially impacting. Variables with a p-value below 0.2 were removed from the model. Nevertheless, variables recognised as being clinically relevant will not be discarded regardless of the outcome of the test.
- Tests for multi-collinearity among the explanatory variables, to avoid overfitting: A PROC REG was calculated for all variables and the variance inflation factor (VIF) option in the MODEL statement provides the variance inflation factors. If VIF is higher than 10 for two variables, only one should be included in the PSM calculation.
- Validation of the final model, by checking for outliers and examining the propensity score values (e.g., histogram).

Propensity score matching

Greedy 5 to 1 digit matching without replacement was applied. A calliper of 0.025 was used; this corresponded to the maximum score difference used to match two patients. The matching starts with the smallest population and matches 1:1 to the larger cohort. The best match was used in each step.

The variable in the PSM might be changed if all responses have the same value for a strata, e.g., if there are zero patients with a strange condition before baseline, then this variable was dropped in the PSM. A test between the two match populations was then performed to compare UTIs.

Inverse probability weighting

Inverse probability weighting is related to propensity scores, as we used the conditional probabilities as weights on the population. This weighting is done to create a sample, where the distribution of the measured covariates is independent of assigned treatment. The weight of each patient is equal to the inverse of the probability of being assigned the treatment they received (i.e., the inverse propensity score) A regression model was then built with the weights from the above-mentioned approach.

Supplementary Table 1. Codes for the definition of an ssUTI (https://icd.who.int/browse10/2019/en#, and https://www.whocc.no/atc_ddd_index/).

| Type | Codes |
| --- | --- |
| Diagnosis (ICD-10 codes) | N10 Acute tubulo-interstitial nephritis  N11 Chronic tubulo-interstitial nephritis  N12 Tubulo-interstitial nephritis, not specified as acute or chronic  N15 Other renal tubulo-interstitial diseases  N16.0 Renal tubulo-interstitial disorders in infectious and parasitic diseases classified elsewhere  N30.0 Acute cystitis  N30.3 Trigonitis  N30.8 Other cystitis  N30.9 Cystitis, unspecified  N34.0 Urethral abscess  N34.1 Nonspecific urethritis  N34.2 Other urethritis  N39.0 Urinary tract infection, site not specified  N41 Inflammatory diseases of prostate  T83.5 Infection and inflammatory reaction due to prosthetic device, implant and graft in urinary system |
| Antibiotic treatment (ATC codes) | J01XX01 fosfomycin-trometamol  J01CA08 pivmecillinam:  Fluoroquinolone:   - J01MA02 ciprofloxacin - J01MA01 ofloxacin   J01XE01 nitrofurantoin  J01EA trimethoprim  J01EE01 sulfamethoxazole + trimethoprim  J01DB01 cephalexin  J01DD04 ceftriaxone |
